# Are Introduced Species Better Dispersers Than Native Species? A Global Comparative Study of Seed Dispersal Distance

**DOI:** 10.1371/journal.pone.0068541

**Published:** 2013-06-20

**Authors:** Habacuc Flores-Moreno, Fiona J. Thomson, David I. Warton, Angela T. Moles

**Affiliations:** 1 Evolution & Ecology Research Centre, School of Biological, Earth and Environmental Sciences, the University of New South Wales, Kensington, New South Wales, Australia; 2 Landcare Research, Lincoln, New Zealand; 3 Evolution & Ecology Research Centre, School of Mathematics and Statistics, the University of New South Wales, Sydney, New South Wales, Australia; University of Marburg, Germany

## Abstract

We provide the first global test of the idea that introduced species have greater seed dispersal distances than do native species, using data for 51 introduced and 360 native species from the global literature. Counter to our expectations, there was no significant difference in mean or maximum dispersal distance between introduced and native species. Next, we asked whether differences in dispersal distance might have been obscured by differences in seed mass, plant height and dispersal syndrome, all traits that affect dispersal distance and which can differ between native and introduced species. When we included all three variables in the model, there was no clear difference in dispersal distance between introduced and native species. These results remained consistent when we performed analyses including a random effect for site. Analyses also showed that the lack of a significant difference in dispersal distance was not due to differences in biome, taxonomic composition, growth form, nitrogen fixation, our inclusion of non-invasive introduced species, or our exclusion of species with human-assisted dispersal. Thus, if introduced species do have higher spread rates, it seems likely that these are driven by differences in post-dispersal processes such as germination, seedling survival, and survival to reproduction.

## Introduction

It has often been suggested that introduced and/or invasive species have greater natural dispersal abilities than do native or less invasive species [1–4]. For instance Murray and Phillips [1] state that “naturalized invasive species that spread by seed possess enhanced strategies for seed dispersal that are either absent or at least not as prevalent in naturalized species that are not invasive”. Thompson and Davis [5] state that research on traits of invasive plants “has revealed that, when compared with natives or non-invasive aliens, invasive aliens …produce more seeds that are better dispersed…”, and Ordonez and Olff [4] state that “ *analyses comparing regional and global species pools of natives and aliens have found that aliens …*. *produce more seeds that are better dispersed*…”. The idea that introduced species disperse their seeds better than natives species underpins much of our understanding of the dynamics of introduced species, including rates of spread [6–8], range sizes [6,7,9] and the ability of introduced species to take advantage of colonization opportunities arising from disturbance and/or an increase in resource levels [10–13]. In this study, we provide the first general test of the fundamental idea that introduced plant species achieve greater dispersal distances under natural conditions than do native species.

The empirical evidence underlying the idea that introduced species have longer seed dispersal distances than do native species is mixed, based on data for relatively few species, and often relies on indirect measures of dispersal capacity (e.g. size of dispersal structures, terminal velocity or buoyancy). For example, some studies have found that introduced species are more likely to have long-distance dispersal vectors, like humans [14], wind [15] and vertebrates [15]. However other studies have found the contrary, with introduced species less likely to be dispersed by vertebrates [16], no difference in the proportion of expanding natives and invasive introduced species that are wind dispersed [17] or that introduced species are dispersed by human vectors, but not by wind or animal vectors [18]. The few studies that have compared actual dispersal distances between pairs or small sets of introduced and co-occurring native species have also found conflicting results, reporting introduced species to have greater seed dispersal distances than native species [19], or no significant difference between introduced and native species [20,21].

Previous attempts to generalize across local/taxon specific studies have used vote counting (qualitative comparison of the number of studies showing significant and non-significant differences [3,22]), finding a weak trend towards greater seed dispersal capacity for introduced invasive species. Unfortunately, the number of studies included in each review was less than eight, no statistical comparison of the dispersal advantage of introduced over native species was done, and some of the studies analyzed in these reviews inferred dispersal ability from dispersal related traits. Thus, a global, quantitative test of the hypothesis that introduced species have greater seed dispersal distances than do native species was urgently needed. This was our main aim.

Our first step was to run a simple comparison of the dispersal distances achieved by introduced and native species. This comparison will help us to understand the role of dispersal distance in the generation of spatial patterns and species composition in plant communities. For example, differences in dispersal can contribute to differences in relative abundance and species richness between plant communities [23]. Differences in seed dispersal distances can also lead to differences in post-dispersal processes such as seed mortality, germination and seedling survival [6,24]. Plants with short seed dispersal distances are thought to be restricted by greater density-dependent mortality, while plants dispersing further are more restricted by the lack of reproductive partners or the lack of suitable microhabitats [25]. Thus, differences in seed dispersal distance can lead to differences in selective pressure and therefore differences in life histories between introduced and native species.

Seed mass, plant height and dispersal syndrome are crucial ecological traits [26–32] that have been shown to affect the dispersal of plant species [24,33], and which sometimes differ between native and introduced species ( [14–16,18,34–39] but see 17,40). Differences in plant height, seed mass and/or dispersal syndrome could mask or artificially generate a difference in dispersal distance between native and introduced species. We therefore asked a) whether there were significant differences in seed mass, plant height and dispersal syndrome between the native and introduced species in our dataset, and b) whether there were differences in dispersal distance between introduced and native plants once we had accounted for plant height, seed mass and dispersal syndrome. Determining whether the differences in dispersal distance remain significant after accounting for these factors will allow us to determine whether any greater dispersal ability of introduced species arises as part of a coordinated suite of life history traits, or whether the dispersal abilities of introduced species might result from selection on aspects of seed morphology, such as wing/pappus size. Our results will also further our understanding of the suite of traits that distinguish introduced from native species.

In summary, the hypotheses we address are:

1. Introduced species will have greater dispersal distances than do native species.
2. Introduced species will have greater dispersal distances than do native species once plant height, seed mass and dispersal syndrome have been accounted for.

## Materials and Methods

### Ethics statement

All data in this study were extracted from published sources, hence no permission or approval for obtaining the data was required.

### Data collection

We began with the seed dispersal database generated by Thomson et al. [33]. These data were primarily for native species, and there is no similar database for introduced species. We therefore performed a search on ISI Web of Knowledge for papers published between 1906 and 2010 with information on seed dispersal distance of introduced plants. The search terms we used were ‘seed’ + ‘dispersal distance’ or ‘seed dispersal’ + ‘distance’, and ‘dispersal kernel’, ‘dispersal curve’ or ‘seed shadow’ restricted by the terms ‘weed$’, ‘introduced’, ‘invasive’,’non-invasive’, ‘naturaliz*’, ‘alien’, ‘non-native’, and ‘noxious’. We also searched for relevant papers in the reference lists of focal papers. We only included papers that presented observational or experimental information on the mean and/or maximum dispersal distance of plant species under field conditions. This included studies that used seed traps, tracked individual seeds, marked and recaptured seeds and estimated dispersal distances based on tracking vectors and calculating gut or fur retention times. We excluded all studies that estimated seed dispersal distances from seed size, including mass and shape, or dispersal syndrome because these variables were included in the analyses. We excluded studies that used artificial or unrealistic conditions, such as wind tunnels, artificial fur or artificial seeds. Studies that calculated seed dispersal distance from buoyancy tests or terminal velocity tests were also excluded. Studies that estimated dispersal distances based on spatial population mathematical models and inverse modeling of seedling distance to nearest adult or mother plant were excluded because they are influenced by post-dispersal processes such as germination success, seed predation and seedling predation. Studies with less than ten replicates for a given species for either mean or maximum dispersal distance were also excluded. When observational and experimental information were both included in the study, we preferentially used observational information.

Information on species’ status (native/introduced) was extracted from the same papers as dispersal distance data. Information on dispersal distances were extracted from three sources (in diminishing order of preference): 1) tables, 2) main text or 3) graphs using DatathiefIII [41]. Maximum and mean dispersal distances were used instead of percentiles since these were the most common measures throughout the literature.

When possible, information on seed mass and maximum plant height or seed release height was extracted from the same papers as dispersal distance data. Information on seed mass and plant height were extracted from the same three sources as dispersal distance data: 1) tables, 2) main text or 3) graphs using DatathiefIII [41]. Otherwise, information on seed mass and plant height were taken from Moles et al. [26,42], the Royal Botanic Gardens, Kew’s Seed Information Database [43] or Mason et al. [40]. For each species we used maximum plant height where possible, but where these data were not available we used the maximum recorded mean plant height. We extracted information on dispersal syndrome from the same papers as dispersal distance data. Dispersal syndrome information was only extracted when the paper explicitly stated that the dispersal distance data were associated with a given dispersal syndrome. Species were initially grouped into four dispersal syndromes: wind, water, animal and unassisted. However, our sample size for water dispersal was very low, and since mean dispersal distance of water and wind dispersed species do not significantly differ (*P* = 0.70) they were treated as one category (water/wind syndrome) to increase our statistical power. In the case of maximum dispersal distance water-dispersed species were excluded because of scarce data (n = 8). Therefore only three categories were used in final analyses. Data for species with more than one dispersal syndrome were included as separate data points for each syndrome.

In total our database contained information on 411 species from 92 families, including data for 360 species in their home range and 51 species in their introduced range. Of the 51 introduced species in our study, five (12%) were classified as naturalized (species that establish self-replacing populations without range expansion; sensu [44]) and 46 (88%) as invasive (introduced species with rapid population increase and range expansion; sensu [44]). Introduced species represented 12.4% of our seed dispersal distance database. Although this is a modest proportion of the whole dataset, it follows the trend reported by Vitousek et al. [45], where on average introduced species represent 8.3% of large continental area floras (e.g. Europe, Tropical Africa, Chile) and 13.3% of smaller continental area floras (e.g. Egypt, Queensland, Texas).

Dispersal distance, seed mass and plant height data were log_10_-transformed before analysis.

### Data analyses

We began by asking whether there were differences in dispersal distance, plant height, seed mass and dispersal syndrome between native and introduced species. We compared the dispersal distances, heights and seed masses of native and introduced species with Student’s *t*-tests, assuming unequal variance. To compare dispersal syndrome between native and introduced species, we used a contingency table. Analyses for dispersal syndrome were performed both with all available data, and excluding water-dispersed species for which we had a very small sample size for introduced species (n = 4). Results were consistent, and for brevity, we present only the more robust analysis based on the three well-replicated dispersal syndromes.

Next, we asked whether there were differences in mean and maximum dispersal distance when accounting for plant height, seed mass or dispersal syndrome individually. For seed mass and plant height, we used ANCOVAs in which introduced/native status was our categorical variable, seed mass or plant height our covariates, and mean or maximum dispersal distance was the dependent variable. We began by confirming that our data fulfilled the homogeneity of variance assumption for ANCOVA (Figure S1). ANCOVAs included terms for species’ status, a trait covariate (plant height or seed mass) and their interaction (status × trait). A significant effect of species’ status would show different average dispersal distances between status, for a given value of the trait covariate. A significant difference in the trait covariate (seed mass or plant height) would show a slope ≠ 0, for species of a given status. A significant effect of the interaction between status and the trait variable would show a difference in the relationship between dispersal distance and the trait covariate, between introduced and native species. That is, introduced and native species would have different slopes and intercepts at the same time. To test whether introduced and native species differ in dispersal distance once the effect of dispersal syndrome had been accounted for we ran a linear model where species’ status and dispersal syndrome were our predictor variables and mean or maximum dispersal distances were our response variables.

We ran a linear model to test whether dispersal distance differs between species as an effect of their native or introduced status once dispersal syndrome, seed mass and plant height had been accounted for. For these linear models our predictor variables were species’ status (native or introduced), dispersal syndrome, seed mass and plant height, and our dependent variables were maximum and mean dispersal distance.

### Data considerations

We did not have enough data (and thus degrees of freedom) to explicitly control for every possible correlate of dispersal distance. We therefore selected a few particularly relevant traits (seed mass, plant height and dispersal syndrome) to include in the main analyses. However, there are other ecological variables that could have an important effect on dispersal distance. We therefore concluded with a series of analyses that explored the potential effects of site to site variation, biome, taxonomy, growth form, ability to fix nitrogen, introduced species’ level of invasiveness (naturalized vs. invasive species) and human assisted dispersal.

The majority of our analyses were run as linear models. However, some datapoints came from the same sites, and so are not fully independent. To address this, we ran models including a random effect for site. These analyses cannot be run with missing values, so first we generated and reanalyze a subset of data with no missing values. The results were broadly consistent with previous results (Table S3). Then, we reanalyze the subset of data with no missing values including a random effect for site (Supporting Information S1). Information on the number of missing values for seed mass, plant height and dispersal syndrome data are available in Table S4.

We tested whether the dispersal distance of introduced and native species differed once the effect of biome (tropical forest, temperate forest, grassland, shrubland, woodland and other; Table S1) had been accounted for using a linear model where biome and species’ status (introduced and native) where our predictor variables and mean or maximum dispersal distance were our dependent variables.

Our next step was to calculate the taxonomic distribution of our study species (Table S1). We used a Yates’ Chi-square because of the high proportion of cells in the analysis with expected values less than five [46]. We did not perform phylogenetic contrast analyses because neither species’ status, nor dispersal distance are heritable traits. Although certain taxa are more likely to be introduced [47,48], a plant doesn’t evolve to be introduced. Dispersal distance is affected by heritable traits such as seed mass, plant height and dispersal syndrome (which we consider in our analyses), but dispersal distance is also affected by non-heritable factors such as the characteristics of the surrounding landscape, wind conditions, the availability of dispersers, and a good deal of chance [32,49];. As phylogenetic analyses are explicitly evolutionary (for instance, Felsenstein’s 1985 [50] method assumes that traits evolve under Brownian motion along branches), phylogenetic analyses are not appropriate for our data.

We used contingency tables to test whether there was a difference in the proportion of introduced and native species with a given growth form (woody vs. non-woody) and with or without nitrogen-fixing capacity.

Many of the predictions about the differences between introduced and native species, and the studies that test these predictions are phrased broadly to include all introduced species [4,35,38,51,52]. However, other predictions are about the difference between native species and invasive introduced species. Only a small proportion of introduced species become invasive [53]. Our main analyses allow us to address the broader question of whether introduced species differ functionally from native species (e.g. [35,54,55]) and if they do, to identify which traits or characteristics are associated with species that have become established in new environment (e.g. [4,56,57]). However, we ran ANOVA tests comparing native species to a subset of invasive introduced species (n = 46), to determine whether the patterns we observed across the full dataset hold up when non-invasive introduced species were excluded. Our predictor variable was species status (native or introduced) and our dependent variables were mean or maximum dispersal distance.

Transport of seeds by humans (for example, by harvesters or mowing machines) was excluded from the main analyses because it is clearly a different process to natural seed dispersal. However, to test whether the inclusion of human-transported species had any effect on our results we ran ANOVA tests including human-dispersed species (two native and five introduced). In these analyses our predictor variable was species’ status and the dependent variable was mean or maximum dispersal distance.

All analyses were performed in R [58], with species as the replicates. We report partial R^2^s throughout. Species’ status (introduced vs. native) is a predictor variable in analyses, rather than the dependent. That is, our analyses ask whether native and introduced species differ in dispersal distance, rather than using dispersal distance to predict whether a species will be introduced or not.

## Results

**Figure 1:**
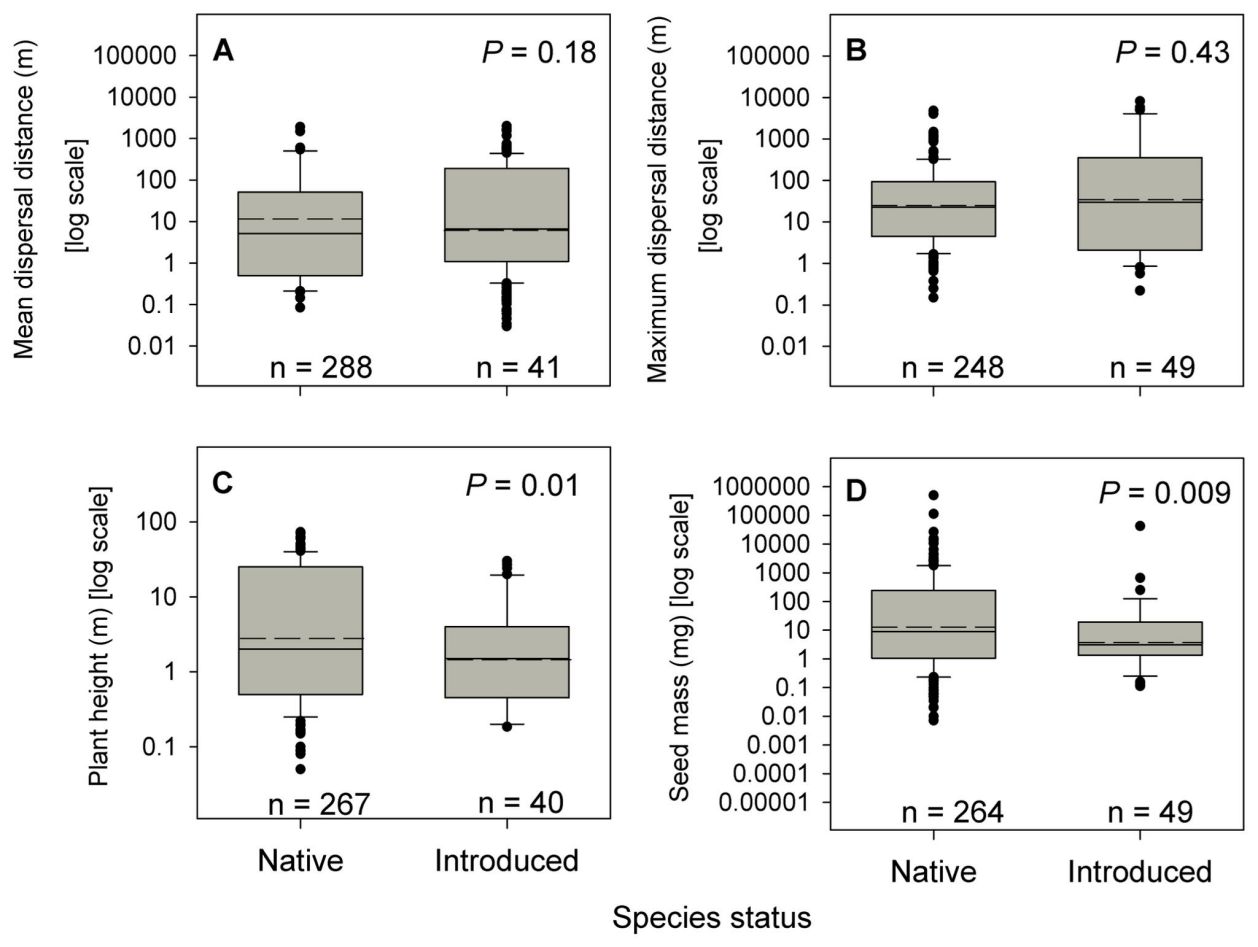
Comparison of native and introduced species’ dispersal distances, plant height and seed mass. Black dashed lines represent mean values. The boxes represent the 25^th^, 50^th^ and 75^th^ percentiles. Whiskers represent the 10^th^ and 90^th^ percentiles, outliers are represented as points. Sample sizes are number of species.

Contrary to our expectations, we found no significant difference between native and introduced species’ mean (*P* = 0.18, Figure 1A) or maximum seed dispersal distances (*P* = 0.43; Figure 1B).

Native plant species were approximately twice as tall as were introduced species (*P* = 0.01, Figure 1C) and had seeds three times bigger (*P* = 0.009, Figure 1D) than did introduced plant species. The proportion of introduced and native species using a given dispersal syndrome was also significantly different (Χ^2^ = 42.43, d.f. = 2, *P* < 0.0001). There were significantly more introduced (53.4%) than native (21.7%) species with wind- or water-dispersed seeds. There were also significantly more introduced (29.3%) than native species (16.1%) with unassisted dispersal. Finally, there was a significantly higher proportion of native species (62.2%) than introduced species (17.2%) with animal dispersal (Table S1).

As expected, seed mass, plant height and dispersal syndrome all affected dispersal distance. Seed mass had a significant positive main effect on both mean (*P* < 0.0001) and maximum (*P* = 0.005) dispersal distance, and dispersal distance increased with increasing plant height in all analyses (*P* < 0.0001). On average, animal-dispersed species had the highest mean (33.4 m) and maximum dispersal distance (51.5 m), followed by water/wind-dispersed species for mean dispersal distance (3.1 m) and wind-dispersed species for maximum dispersal distance (22.04 m). Species with unassisted dispersal had the lowest dispersal distance of all syndromes for both mean (0.7 m) and maximum (2.5 m) dispersal distance.

When we accounted for the effect of one plant trait (seed mass, plant height or dispersal syndrome), we tended to find significant interactions between species status and the plant trait under consideration (Figure S1 and Table S2). For mean and maximum dispersal distance we found a significant interaction between plant height and species status (*P* = 0.03 and < 0.0001 respectively). That is, differences in dispersal distance between introduced and native species vary with plant height. There were no significant differences between introduced and native species’ mean dispersal distance after accounting for the effect of seed mass (P = 0.43) or dispersal syndrome (*P* = 0.48). However, for maximum dispersal distance there were significant interactions between seed mass and species’ status (*P* = 0.05) and between dispersal syndrome and species’ status (*P* = 0.003). That is, the difference in maximum dispersal distance between introduced and native species varies with seed mass or dispersal syndrome.

To determine whether species’ status affected dispersal distance after accounting for variation due to seed mass, plant height, and dispersal syndrome simultaneously, we constructed a model including terms for status, dispersal syndrome, plant height and seed mass and interactions. For mean dispersal distance, none of the five interactions between species’ status seed mass, plant height and/or dispersal syndrome were significant (*P* > 0.18; Table 1), and the main effect of status was not significant (*P* = 0.67; Table 1). That is, once we have accounted for the effect of seed mass, plant height and dispersal syndrome, there is no significant difference in mean seed dispersal distance between native and introduced species. Interestingly, height (R^2^ = 0.23) explained almost six times as much variation as did dispersal syndrome (R^2^ = 0.04), which was the next best predictor of mean dispersal distance, and seed mass (R^2^ = 0.02) and species’ status (R^2^ < 0.001) made much smaller contributions to the predictive power of the model. The overall model explained 63% of the variation in mean dispersal distance, a remarkable outcome given that our data come from a range of taxa in a broad range of ecosystems worldwide.

**Table 1:** Effect of species’ status (native vs. introduced), seed mass, plant height and dispersal syndrome (animal, unassisted, water/wind), and their interactions on mean dispersal distance.

**Term**	**Sum of Squares**	***F* ratio**	***P***
Status	0.08	0.18	0.67
Seed mass	2.85	6.61	0.01
Plant height	30.68	71.33	< 0.0001
Dispersal syndrome	5.29	6.15	0.003
Status × Dispersal syndrome	0.15	0.17	0.85
Status × Seed mass	0.27	0.63	0.43
Status × Plant height	0.12	0.27	0.60
Status × Plant height × Dispersal syndrome	2.68	1.56	0.19
Status × Seed mass × Dispersal syndrome	2.11	1.23	0.30

We next constructed a model for maximum dispersal distance including terms for status, dispersal syndrome, plant height and seed mass and interactions. There were two significant three-way interactions, and two significant two-way interactions, all including status (Table 2). That is, the effect of status differs according to height, seed mass and dispersal syndrome. As in the model for mean dispersal distance, the main effect of species’ status was not significant (*P* = 0.23). However, most statisticians advise against interpreting main effects in the presence of significant higher order interactions [59].

**Table 2:** Effect of species’ status (native vs. introduced), seed mass, plant height and dispersal syndrome (animal, unassisted, water/wind), and their interactions on maximum dispersal distance.

**Term**	**Sum of Squares**	***F* ratio**	***P***
Status	0.58	1.43	0.23
Seed mass	1.52	3.73	0.06
Plant height	3.35	8.21	0.005
Dispersal syndrome	23.09	28.31	< 0.0001
Status × Dispersal syndrome	0.25	0.31	0.74
Status × Seed mass	4.37	10.72	0.001
Status × Plant height	3.60	8.82	0.003
Status × Plant height × Dispersal syndrome	8.46	5.19	<0.001
Status × Seed mass × Dispersal syndrome	8.08	4.95	<0.001

### Data considerations

1. Site to site variation.

Analyses including a random effect for site (Supporting Information S1) were broadly consistent with previous analyses. That is, the fact that species occurring at the same site are not fully independent is not the reason for a lack of a significant difference in the dispersal distance of introduced and native species.

2. Biome.

The main effect of species status (native vs. introduced) was not significant for either mean or maximum dispersal distance once biome had been accounted for (*P* = 0.07 and 0.29, respectively). There was a significant interaction between biome and species’ status for maximum dispersal distance (*P* = 0.0007). That is, the maximum dispersal distance achieved by introduced and native species varies by biome. However, there is not a consistent difference in maximum dispersal distance between introduced and native species after accounting for the effect of biome.

3. Taxonomy.

Although there were some modest differences in the relative representation of different taxonomic groups, between native and introduced species (Table S1), these differences were not significant (Yates’ χ^2^ = 14.71, d.f. = 9, *P* = 0.10).

4. Growth form.

A significantly higher proportion of native species than introduced species had a woody growth form (Χ^2^ = 18.55, d.f. = 1, *P* < 0.0001; Table S1). However, when we ran a linear model with species’ status, plant height, seed mass and growth form as predictor variables, neither growth form (*P* > 0.20) nor species’ status (*P* > 0.66) had a significant effect on mean or maximum dispersal distance.

5. Ability to fix nitrogen.

There was no significant difference in the proportion of introduced and native species that are N-fixers (Yates Χ^2^ = 0.09, d.f. = 1, *P* = 0.77; Table S1).

6. Naturalized vs. invasive species.

An analysis of the differences between species with varying levels of invasiveness is outside of the scope of our present study. However, only five of our introduced species were not invasive. Excluding these five species from analysis did not qualitatively affect our results. There was no significant difference in the mean (*P* = 0.14) or maximum (*P* = 0.63) seed dispersal distance when the non-invasive species were excluded from analysis.

7. Human-assisted dispersal.

There was no significant difference between the mean (*P* = 0.23) or maximum (*P* = 0.25) seed dispersal distance of introduced and native species when the seven human-dispersed species were included in the analysis. That is, including species with human-assisted dispersal did not qualitatively affect our results.

In summary, the lack of a significant difference in average dispersal distance between native and introduced species is not due to a relationship being obscured by differences in the biomes from which the data were taken, the taxonomic composition of the datasets, the growth form of the species, or differences in the proportion of native vs. introduced species that were able to fix nitrogen. We can also exclude the possibility that our inclusion of species with different levels of invasiveness, our exclusion of species with human-assisted dispersal, or our treatment of replicate species from the same sites has masked a significant difference in seed dispersal distance between introduced and native species.

## Discussion

While there are some invasive plants that have truly spectacular rates of spread across the landscape (e.g. *Pueraria* *lobata* in the United States or *Abutilon* *theophrasti* in Europe), our data show that there is no overall difference in dispersal distances achieved by native and introduced species. This pattern holds after accounting for variation due to traits such as seed mass, plant height and dispersal syndrome. Our findings overturn traditional assumptions about the importance of dispersal distance for the spread of introduced species [2,6,17,60], and have two implications for management. First, there is no fundamental reason to expect native species to be more limited by dispersal ability when responding to global change than are introduced species. Second, we need to remember that introduced species, like native species, have a range of traits, and do not all have exceptional dispersal abilities.

There are often differences in dispersal-related traits between native and introduced/invasive species [35,61–63]. It is clearly important to ask whether there are differences in the dispersal distances achieved by native vs. introduced species when all else is equal (that is, when comparing similar types of species). However, the important variable determining rates of spread of introduced and native species is not dispersal distance after accounting for other variables, but simply how far the seeds of each group of species travel. That is, if introduced species did differ in a trait with native species, and this helped their seeds to disperse greater distances, the end results would still be a greater rate of spread for introduced species.

Although seed dispersal is an important determinant of plant distributions, getting there is just part of the struggle, and seed arrival does not necessarily translate to recruitment [6]. Thompson and Davis [5] state that distinction between plant winners or losers “often owes rather little to native or alien status”. In the case of dispersal distance this is true. Species’ status explained less than 1% of the variation in both mean and maximum dispersal distance. To fully understand introduced species’ high spread rates and rapid colonization we need to consider other factors that could favor rapid spread; these may include short times to reproduction, high post dispersal survival, high germination, human mediated processes or propagule pressure [52,64–66]. Clearly, the contribution of human-mediated dispersal and post-dispersal mechanisms is an area of invasion biology where future research is urgently needed in order to understand the patterns and processes that govern the spread of introduced species.

Not all introduced species are invasive. We wondered whether over representation of non-invasive introduced species (naturalized species sensu Richardson [44]) in our data set could have obscured differences in dispersal distance between introduced and native species. However, of the 51 introduced species included in our study, only five were classified as naturalized by the primary studies or environmental agencies of the countries where the studies were done, while 46 were classified as invasive. Thus, a lack of invasive species is not responsible for the lack of a significant difference between dispersal distance in native and introduced species. If anything, the predominance of invasive introduced species in our dataset would be expected to bias our study towards finding a significant difference in dispersal ability between introduced and native species, particularly since ecologists may be more likely to study seed dispersal on introduced species that have relatively rapid rates of spread.

We still have a great deal to learn about the factors underlying the success of introduced species, and our study highlights several important questions for the future. First, higher recruitment success could play a major role in facilitating the establishment and rapid population growth rate of introduced species [3,52,67]. Thus, determining whether introduced species have higher recruitment success under natural conditions is a promising direction. Second, species with different levels of invasiveness might differ in their traits and life history characteristics [68,69]. Quantifying the differences between species with different levels of invasiveness could help us identify which traits or trait values are most strongly associated with the success of invasive species [68,69]. Finally, using formal path analyses we could determine the extent to which observed differences in fundamental ecological traits between introduced and native species actually translate to differences in recruitment and rates of spread. That is, we could test the adaptive nature of the morphological and functional traits associated with introduced and/or invasive species.

The idea that introduced species, particularly invasive species, are better dispersers than are native species was based on the observation of high spread rates, high population growth rates and broad differences in dispersal related traits that theoretically would give introduced species great advantage in achieving high dispersal distances [7–9,40]. However, our data are not consistent with this idea. Our findings reshape and advance our knowledge of the ecological characteristics and mechanisms that underlie the spatiotemporal dynamics of introduced species. These concepts are not only central to understanding the ecology of introduced species, but are also central to their management.

## Supporting Information

Figure S1 Graphs of relationships between dispersal distance of introduced vs. native species when accounting for seed mass, plant height or dispersal syndrome individually. (DOC)

Supporting Information S1 Supporting Information S1. Analyses including a random effect for site.
(DOC)

Table S1 Attributes of introduced and native species. (DOC)

Table S2 Details of analyses of dispersal distance of native vs. introduced species when accounting for seed mass, plant height or dispersal syndrome, individually. (DOC)

Table S3 Comparisons of introduced and native species’ seed dispersal distances using a subset of data with no missing values. (DOC)

Table S4 Total number of missing values for trait data. (DOC)
