# Supplementary material for: Are Introduced Species Better Dispersers Than Native Species? A Global Comparative Study of Seed Dispersal Distance
Source: PLoS One. 2013 Jun 20;8(6):e68541. doi: 10.1371/journal.pone.0068541 (PMC3688602; doi:10.1371/journal.pone.0068541)
Supplement: Figure S1 — (DOC) [file pone.0068541.s001.doc]

**Figure S1: Graphs of relationships between dispersal distance of introduced vs. native species when accounting for seed mass, plant height or dispersal syndrome individually**.

1) Black circles and dashed lines represent introduced species, while white circles and complete lines represent native species. *P*_s_ represents the *P* values of the species status effect on the model. *P*_int_ represents the *P* value of the interaction of seed mass and species status (panels A and B) or plant height and species’ status (panels C and D) effect on the model.

2) Differences between native and introduced species’ seed dispersal distances for species with animal dispersal, wind/water dispersal or unassisted dispersal. Black dashed lines represent mean values. The boxes represent 25^th^, 50^th^ and 75^th^ percentiles. Whiskers represent the 10^th^ and 90^th^ percentiles, outliers are shown as points. Letters above boxes represent dispersal syndromes with significantly (P < 0.05) different dispersal distances. Numbers below boxes are sample sizes. *P*_s_ represents the *P* values of the species status effect on the model. *P*_int_ represents the *P* value of the interaction of dispersal syndrome and species’ status on the model.

3) Residuals vs. fitted values plots for the mean dispersal distance comparison of introduced and native species accounting for plant height (A) and seed mass (B), and residuals vs. fitted values plots for the maximum dispersal distance comparison of introduced and native species accounting for plant height (C) and seed mass (D). Note the lack of a well-defined pattern in the four plots suggesting homogeneity of variance, and thus the suitability of these data for ANCOVA analysis.
