# Supplementary material for: Are Introduced Species Better Dispersers Than Native Species? A Global Comparative Study of Seed Dispersal Distance
Source: PLoS One. 2013 Jun 20;8(6):e68541. doi: 10.1371/journal.pone.0068541 (PMC3688602; doi:10.1371/journal.pone.0068541)
Supplement: Supporting Information S1 — Analyses including a random effect for site. (DOC) [file pone.0068541.s002.doc]

**Supporting Information S4. Analyses including a random effect for site.**

To test the influence of site on the dispersal distance comparison between introduced and native species we ran a linear mixed model were our predictor variables were plant height, seed mass, dispersal syndrome and a random term for site and our dependent variables were mean and maximum dispersal distance. Analyses were done on log-transformed dispersal distance, seed mass and plant height data. Plant height explained 1.2 times more variation than did dispersal syndrome, and almost twice as much variation as did seed mass (Table S4.1). Species’ status explained approximately five times less variation in mean dispersal distance than did plant height (Table S4.1). The term that explained the most variation in maximum dispersal distance was dispersal syndrome, followed by plant height, seed mass and the interaction between dispersal syndrome and species’ status (Table S4.2). Species’ status explained eleven times less variation in maximum dispersal distance than did dispersal syndrome (Table S4.2).

**Table S4.1**. **Comparison between introduced and native species’ mean dispersal distance including a random effect for site.**

| **Source** | **Estimate** | ***P*** |
| --- | --- | --- |
| Species status | 0.16 | 0.99 |
| Seed mass | -0.05 | 0.36 |
| Plant height | 0.63 | < 0.0001 |
| Unassisted dispersal syndrome | -0.41 | 0.02 |
| Wind/water dispersal syndrome | -0.11 | 0.44 |
| Species status × Unassisted dispersal syndrome | -0.18 | 0.99 |
| Species status × Wind/water dispersal syndrome | -0.18 | 0.99 |
| Species status × Seed mass | -3.14 | 0.87 |
| Species status × Plant height | 7.33 | 0.85 |
| Native status × Seed mass × Unassisted dispersal syndrome | 0.09 | 0.42 |
| Native status × Seed mass × Wind/water dispersal syndrome | -0.33 | 0.02 |
| Introduced status × Seed mass × Unassisted dispersal syndrome | 3.22 | 0.87 |
| Introduced status × Seed mass × Wind/water dispersal syndrome | 3.54 | 0.86 |
| Native status × Plant height × Unassisted dispersal syndrome | -0.17 | 0.43 |
| Native status × Plant height × Wind/water dispersal syndrome | 0.79 | <0.001 |
| Introduced status × Plant height × Unassisted dispersal syndrome | -7.16 | 0.86 |
| Introduced status × Plant height × Wind/water dispersal syndrome | -6.37 | 0.87 |

**Table S4.2** **Comparison between introduced and native species’ maximum dispersal distance including a random effect for site.**

| **Source** | **Estimate** | ***P*** |
| --- | --- | --- |
| Species status | -0.72 | 0.75 |
| Seed mass | -0.01 | 0.62 |
| Plant height | 0.53 | <0.0001 |
| Unassisted dispersal syndrome | -0.54 | 0.001 |
| Wind dispersal syndrome | -0.05 | 0.73 |
| Species status × Unassisted dispersal syndrome | 0.60 | 0.76 |
| Species status × Wind dispersal syndrome | 0.63 | 0.77 |
| Species status × Seed mass | -10.66 | 0.55 |
| Species status × Plant height | 22.35 | 0.54 |
| Native status × Seed mass × Unassisted dispersal syndrome | 0.06 | 0.40 |
| Native status × Seed mass × Wind dispersal syndrome | -0.48 | <0.001 |
| Introduced status × Seed mass × Unassisted dispersal syndrome | 10.85 | 0.54 |
| Introduced status × Seed mass × Wind dispersal syndrome | 11 | 0.54 |
| Native status × Plant height × Unassisted dispersal syndrome | -0.08 | 0.56 |
| Native status × Plant height × Wind dispersal syndrome | 0.62 | 0.005 |
| Introduced status × Plant height × Unassisted dispersal syndrome | -22.10 | 0.54 |
| Introduced status × Plant height × Wind/water dispersal syndrome | -21.37 | 0.55 |
