# Supplementary material for: Are Introduced Species Better Dispersers Than Native Species? A Global Comparative Study of Seed Dispersal Distance
Source: PLoS One. 2013 Jun 20;8(6):e68541. doi: 10.1371/journal.pone.0068541 (PMC3688602; doi:10.1371/journal.pone.0068541)
Supplement: Table S1 — (DOC) [file pone.0068541.s003.doc]

**Table S1. Attributes of introduced and native species.**

Observed number (and percent) of introduced and native species in each category for dispersal syndrome, taxonomic group, nitrogen fixation, growth form and biome. A species could be counted in more than one category for dispersal syndrome, but not for any of the other factors. Taxonomy follows the Angiosperm Phylogeny Website . Proteales, Vitales and Gymnosperms (each represented by few species) were grouped to avoid having cells with expected values less than 1, which invalidates the Chi-square test .

|  | **Species’ status** | |
| --- | --- | --- |
| **Dispersal syndrome** | **Native** | **Introduced** |
| Animal | 232 (62.2%) | 10 (17.2%) |
| Unassisted | 60 (16.1%) | 17 (29.3%) |
| Water/Wind | 81 (21.7%) | 31 (53.4%) |
|  |  |  |
| **Taxonomic group** | **Native** | **Introduced (56)** |
| Monocots | 31 (8.6%) | 8 (14.3%) |
| Magnoliids | 34 (9.4%) | 1 (1.8%) |
| Ranunculales | 6 (1.7%) | 2 (3.6%) |
| Core eudicots (Caryophyllales, Saxifragales, Santalales) | 21 (5.8%) | 4 (7.1%) |
| Rosids I (Fabidae) | 116 (32.0%) | 8 (14.3%) |
| Rosids II (Malvidae) | 52 (14.4%) | 10 (17.8%) |
| Basal asterids (Ericales) | 17 (4.6%) | 2 (3.6%) |
| Asterids I (incl Lamiales, Gentianales, Solanales) | 39 (10.8%) | 8 (14.3%) |
| Asterids II (incl Asterales, Apiales) | 33 (9.1%) | 12 (21.4%) |
| Other (Proteales, Vitales, Gymnosperms) | 13 (3.6%) | 1 (1.8%) |
|  |  |  |
| **Nitrogen fixation** | **Native (362)** | **Introduced (56)** |
| Present | 27 (7.5%) | 5 (9.8%) |
| Absent | 333 (92.5%) | 46 (90.2%) |
|  |  |  |
| **Growth form** | **Native** | **Introduced** |
| Woody | 227 (63.1%) | 16 (31.4%) |
| Non-woody | 133 (36.9%) | 35 (68.6%) |
|  |  |  |
| **Biome** | **Native** | **Introduced** |
| Grassland | 81 (22.5%) | 23 (45.1%) |
| Shrubland | 19 (5.3%) | 1 (2%) |
| Temperate forest | 58 (16.1%) | 7 (13.7%) |
| Tropical forest | 146 (40.6%) | 4 (7.8 %) |
| Woodland | 42 (11.7%) | 6 (11.8%) |
| Other/unknown | 14 (3.9%) | 10 (19.6%) |

**References:**
