# Supplementary material for: Are Introduced Species Better Dispersers Than Native Species? A Global Comparative Study of Seed Dispersal Distance
Source: PLoS One. 2013 Jun 20;8(6):e68541. doi: 10.1371/journal.pone.0068541 (PMC3688602; doi:10.1371/journal.pone.0068541)
Supplement: Table S2 — (DOC) [file pone.0068541.s004.doc]

**Table S2.** **Details of analyses of dispersal distance of native vs. introduced species when accounting for seed mass, plant height or dispersal syndrome, individually.**

| **1)** **MEAN SEED DISPERSAL DISTANCE AND SEED MASS** | | | | | |
| --- | --- | --- | --- | --- | --- |
| **Term** | **Estimate** | **Standard error** | **Sum of Squares** | **d.f.** | ***P*** |
| Intercept | 0.3 | 0.09 | 10 | 14 | 0.002 |
| Seed mass | 0.33 | 0.05 | 47.88 | 1 | < 0.0001 |
| Status | 0.18 | 0.23 | 0.65 | 1 | 0.43 |
| Status × seed mass | 0.14 | 0.16 | 0.84 | 1 | 0.37 |
| Residuals |  |  | 243.25 | 233 |  |

**2) MAXIMUM SEED DISPERSAL DISTANCE AND SEED MASS**

| **Term** | **Estimate** | **Standard error** | **Sum of Squares** | **d.f.** | ***P*** |
| --- | --- | --- | --- | --- | --- |
| Intercept | 1.17 | 0.08 | 180 | 1 | < 0.0001 |
| Seed mass | 0.13 | 0.04 | 7.1 | 1 | 0.005 |
| Status | 0.05 | 0.16 | 0.06 | 1 | 0.80 |
| Status × seed mass | 0.28 | 0.1 | 3.58 | 1 | 0.05 |
| Residuals |  |  | 216.46 | 245 |  |

**3) MEAN SEED DISPERSAL DISTANCE AND PLANT HEIGHT**

| **Term** | **Estimate** | **Standard error** | **Sum of Squares** | **d.f.** | ***P*** |
| --- | --- | --- | --- | --- | --- |
| Intercept | 0.24 | 0.06 | 8.12 | 1 | < 0.0001 |
| Plant height | 0.97 | 0.06 | 140.85 | 1 | < 0.0001 |
| Status | 0.15 | 0.15 | 0.57 | 1 | 0.3 |
| Status × plant height | 0.47 | 0.21 | 2.58 | 1 | 0.03 |
| Residuals |  |  | 116.19 | 228 |  |

**4) MAXIMUM SEED DISPERSAL DISTANCE AND PLANT HEIGHT**

| **Term** | | **Estimate** | **Standard error** | **Sum of Squares** | **d.f.** | ***P*** |  | | | |
| --- | --- | --- | --- | --- | --- | --- | --- | --- | --- | --- |
| Intercept | | 1.09 | 0.06 | 195.81 | 1 | < 0.0001 |  | | | |
| Plant height | | 0.46 | 0.07 | 28.07 | 1 | < 0.0001 |  | | | |
| Status | | 0.03 | 0.15 | 0.02 | 1 | 0.86 |  | | | |
| Status × plant height | | 0.83 | 0.21 | 9.24 | 1 | 0.0001 |  | | | |
| Residuals | |  |  | 146.08 | 236 |  |  | | | |
|  | **5) MEAN SEED DISPERSAL DISTANCE AND DISPERSAL SYNDROME**   | **Term** |  |  | **Sum of Squares** | **d.f.** | ***P*** | | --- | --- | --- | --- | --- | --- | | Intercept |  |  | 344 | 1 | < 0.0001 | | Dispersal syndrome |  |  | 104.66 | 2 | < 0.0001 | | Status |  |  | 2.28 | 1 | 0.48 | | Dispersal syndrome × status |  |  | 1.53 | 2 | 0.1 | | Residuals |  |  | 291.68 | 290 |  | | | | | | | |  |  |  |
|  | **6) MAXIMUM SEED DISPERSAL DISTANCE AND DISPERSAL SYNDROME**   | **Term** |  |  | **Sum of Squares** | **d.f.** | ***P*** | | --- | --- | --- | --- | --- | --- | | Intercept |  |  | 397.03 | 1 | < 0.0001 | | Dispersal syndrome |  |  | 50.9 | 2 | < 0.0001 | | Status |  |  | 4.55 | 1 | 0.008 | | Dispersal syndrome × status |  |  | 7.75 | 2 | 0.003 | | Residuals |  |  | 195.55 | 301 |  | | | | | | | |  |  |  |
|  |  | | | | | | |  |  |  |

**7) ESTIMATES FOR DISPERSAL SYNDROME ANALYSES**

|  | **Mean dispersal distance** | | **Maximum dispersal distance** | |
| --- | --- | --- | --- | --- |
| **Term** | **Estimate** | **Standard error** | **Estimate** | **Standard error** |
| Intercept | 1.51 | 0.07 | 1.6663 | 0.0674 |
| Unassisted | -1.7 | 0.15 | -1.1461 | 0.1315 |
| Water/wind | -1.24 | 0.16 | -0.4837 | 0.124 |
| Introduced | 0.27 | 0.39 | 0.6978 | 0.2636 |
| Unassisted × status | -0.42 | 0.49 | -1.1682 | 0.3471 |
| Water/wind × status | 0.41 | 0.46 | -0.4753 | 0.3245 |
