# Supplementary material for: Are Introduced Species Better Dispersers Than Native Species? A Global Comparative Study of Seed Dispersal Distance
Source: PLoS One. 2013 Jun 20;8(6):e68541. doi: 10.1371/journal.pone.0068541 (PMC3688602; doi:10.1371/journal.pone.0068541)
Supplement: Table S3 — (DOC) [file pone.0068541.s005.doc]

**Table S3.** **Comparisons of introduced and native species’ seed dispersal distances using a subset of data with no missing values.**

Status refers to whether a species is native or introduced. All analyses were done on log-transformed data.

| **1) ANALYSES WITH NO ADDITIONAL PREDICTORS** | | | | | | |
| --- | --- | --- | --- | --- | --- | --- |
|  | **Mean dispersal distance** | | | **Maximum dispersal distance** | | |
| **Term** | **t** | **d.f.** | ***P*** | **t** | **d.f.** | ***P*** |
| Status | -0.42 | 41.2 | 0.7 | -0.68 | 47.1 | 0.5 |
| **2) ANALYSES WITH ONE ADDITIONAL PREDICTOR** | | | | | | |
|  | **Mean dispersal distance** | | | **Maximum dispersal distance** | | |
| **Term** | **Sum of squares** | **d.f.** | ***P*** | **Sum of squares** | **d.f.** | ***P*** |
| Status | 0.09 | 1 | 0.7 | 0.25 | 1 | 0.6 |
| Seed mass | 31.6 | 1 | <0.0001 | 4.34 | 1 | 0.02 |
| Status × seed mass | 2.6 | 1 | 0.09 | 2.8 | 1 | 0.06 |
| Residuals | 183.9 | 208 |  | 172.11 | 223 |  |
|  |  |  |  |  |  |  |
| Status | 0.5 | 1 | 0.3 | 0.000 | 1 | 1 |
| Plant height | 101.6 | 1 | <0.0001 | 24.8 | 1 | <0.0001 |
| Status × plant height | 2.7 | 1 | 0.02 | 5.4 | 1 | 0.004 |
| Residuals | 99.8 | 208 |  | 139.3 | 223 |  |
|  |  |  |  |  |  |  |
| Status | 3.06 | 1 | 0.05 | 4.8 | 1 | 0.003 |
| Dispersal syndrome | 52.8 | 2 | <0.0001 | 33.3 | 2 | <0.0001 |
| Status × dispersal syndrome | 2.9 | 2 | 0.2 | 4.5 | 2 | 0.02 |
| Residuals | 157.9 | 206 |  | 114.8 | 215 |  |
| **3) FULL MODEL** | | | | | | |
|  | **Mean dispersal distance** | | | **Maximum dispersal distance** | | |
| **Term** | **Sum of squares** | **d.f.** | ***P*** | **Sum of squares** | **d.f.** | ***P*** |
| Species status | 0.08 | 1 | 0.7 | 0.6 | 1 | 0.2 |
| Seed mass | 2.9 | 1 | 0.01 | 1.5 | 1 | 0.06 |
| Plant height | 30.7 | 1 | <0.0001 | 3.4 | 1 | <0.01 |
| Dispersal syndrome | 5.3 | 2 | <0.01 | 23.1 | 2 | <0.0001 |
| Species status × Dispersal syndrome | 0.15 | 2 | 0.8 | 0.25 | 2 | 0.7 |
| Species status × Seed mass | 0.3 | 1 | 0.4 | 4.4 | 1 | <0.01 |
| Species status × Plant height | 0.12 | 1 | 0.6 | 3.6 | 1 | <0.01 |
| Species status × Plant height × Dispersal syndrome | 2.7 | 4 | 0.2 | 8.5 | 4 | <0.0001 |
| Species status × Seed mass × Dispersal syndrome | 2.1 | 4 | 0.3 | 8.1 | 4 | <0.0001 |
| Residuals | 83.4 | 194 |  | 82.8 | 203 |  |
