# Supplementary material for: Are Introduced Species Better Dispersers Than Native Species? A Global Comparative Study of Seed Dispersal Distance
Source: PLoS One. 2013 Jun 20;8(6):e68541. doi: 10.1371/journal.pone.0068541 (PMC3688602; doi:10.1371/journal.pone.0068541)
Supplement: Table S4 — (DOC) [file pone.0068541.s006.doc]

**Table S4. Present and missing values for trait data.**

Number (and percent) of introduced and native species’ present/missing data for plant height, seed mass and dispersal syndromes. A species could be counted in more than one category for dispersal syndrome, but not for any of the other factors.

**1) Present/missing data for plant height data.**

|  | Species’ status | |
| --- | --- | --- |
| Present/missing data | home | introduced |
| Missing | 93 (25.8%) | 11 (21.6%) |
| Present | 267 (74.2) | 40 (78.4%) |

**2) Present/missing data for seed mass data.**

|  | Species’ status | |
| --- | --- | --- |
| Present/missing data | home | introduced |
| Missing | 96 (26.7%) | 2 (3.9%) |
| Present | 264 (73.3%) | 49 (96.1%) |

**3) Present/missing data for dispersal syndrome data.**

|  | Species’ status | |
| --- | --- | --- |
| Present/missing data | Home | introduced |
| Missing | 0 (0%) | 0 (0%) |
| animal | 232 (62.2%) | 10 (17.2%) |
| unassisted | 60 (16.1%) | 17 (29.3%) |
| Water/wind | 81 (21.1%) | 31 (53.4) |
